# Increasing preoperative cognitive reserve to prevent postoperative delirium and postoperative cognitive decline in cardiac surgical patients (INCORE): Study protocol for a randomized clinical trial on cognitive training

**DOI:** 10.3389/fneur.2022.1040733

**Published:** 2022-12-12

**Authors:** Marius Butz, Rolf Meyer, Tibo Gerriets, Gebhard Sammer, Johanna M. Doerr, Jasmin El-Shazly, Thorsten R. Doeppner, Yeong-Hoon Choi, Markus Schoenburg, Martin Juenemann

**Affiliations:** ^1^Heart and Brain Research Group, Kerckhoff Heart and Thorax Center, Bad Nauheim, Germany; ^2^Department of Neurology, University Hospital Giessen and Marburg, Giessen, Germany; ^3^Cognitive Neuroscience at the Centre of Psychiatry, University Giessen, Giessen, Germany; ^4^Department of Psychology, Justus-Liebig University, Giessen, Germany; ^5^Department of Psychocardiology, Kerckhoff Heart and Thorax Center, Bad Nauheim, Germany; ^6^Department of Cardiac Surgery, Kerckhoff Heart and Thorax Center, Bad Nauheim, Germany

**Keywords:** cardiac surgery, postoperative delirium, postoperative cognitive decline, prehabilitation, cognitive reserve, cognitive training

## Abstract

**Introduction:**

Postoperative delirium (POD) and postoperative cognitive decline (POCD) can be observed after cardiosurgical interventions. Taken together, these postoperative neurocognitive disorders (PNCDs) contribute to increased morbidity and mortality. Preoperative risk factors of PNCD, such as decreased neuropsychometric performance or decreased cognitive daily activities, can be interpreted as reduced cognitive reserve. This study aims to build up cognitive reserves to protect against the development of PNCD through preoperative, home-based, cognitive training.

**Methods:**

The planned research project is a monocentric, two-arm randomized controlled intervention study involving 100 patients undergoing elective cardiac surgery with extracorporeal circulation. Patients will be assigned to a training group or control group. The intervention involves a standardized, paper-and-pencil-based cognitive training that will be performed by the patients at home for ~40 min per day over a preoperative period of 2–3 weeks. The control group will receive neither cognitive training nor a placebo intervention. A detailed assessment of psychological functions will be performed ~2–3 weeks before the start of training, at the end of the training, during hospitalization, at discharge from the acute clinic, and 3 months after surgery. The primary objective of this study is to investigate the interventional effect of preoperative cognitive training on the incidence of POD during the stay in the acute clinic, the incidence of POCD at the time of discharge from the acute clinic, and 3 months after surgery. Secondary objectives are to determine the training effect on objective cognitive functions before the surgery and subjective cognitive functions, as well as health-related quality of life 3 months after surgery.

**Discussion:**

Should it become evident that the use of our cognitive training can both reduce the incidence of POCD and POD and improve health-related quality of life, this intervention may be integrated into a standardized prehabilitation program.

## Introduction

Neuropsychological complications following cardiac surgery include postoperative delirium (POD), postoperative cognitive decline (POCD) (1), and dementia (2). Depending on age and evaluation methods, the frequency of POD after cardiac surgery varies between 14 and 50% (3). The prevalence of POCD is reported to be in the range of 28% between the first and fourth postoperative month and 22% between the sixth and 12th postoperative month (4). Postoperative cognitive improvement (POCI) can also be measured after surgeries but occurs about three to six times less frequently than POCD (5). POCD is more noticeable in objective psychometric assessments and often appears subclinically; nevertheless, patients and their relatives have reported a subjective decrease in patients' cognitive abilities in daily living after heart surgery (6). POD and POCD are related to a reduced quality of life, long-term cognitive decline, increased economic costs, and higher mortality (3).

Preoperative psychological risk factors for the development of POCD include depression (7, 8) and reduced neuropsychometric functions (9). Preexisting cognitive impairment may also contribute to the development of dementia (10). In addition, depression (11), preoperatively reduced objective cognitive functions (11), or reduced cognitive everyday activities such as reading, writing, solving crosswords, and playing computer games (12) could be linked to the development of POD. Furthermore, in a rat model, it was shown that a preoperative enrichment of activity opportunities can lower the rate of POCD (13). In summary, diminished neuropsychometric functions or reduced cognitive everyday activities can be declared as a lower cognitive reserve. In the classical sense, the term “cognitive reserve” refers to the adaptability of cognitive processes, which can help to explain the sensitivity of cognitive abilities or everyday functions in the context of physiological aging and pathological neurodegeneration of the brain. The cognitive reserve can be influenced, among other things, by general cognitive ability in early life (intelligence), education, occupation, physical activity, leisure activities, or social engagement (14). For example, it has been shown that increased cognitive or social leisure activities performed within a wide life span (childhood to adulthood) can reduce the risk of developing dementia (15, 16). Increasing the cognitive reserve through focused cognitive training may, therefore, be a potential intervention target to prevent postoperative neurocognitive dysfunctions.

The primary objective of this study is to investigate the interventional effect of preoperative cognitive training on the incidence of POD during the stay in the acute clinic, the incidence of POCD at the time of discharge from the acute clinic, and 3 months after surgery. Secondary objectives are to determine the training effect on objective cognitive functions before the surgery and subjective cognitive functions, as well as health-related quality of life 3 months after surgery.

## Methods and analysis

### General conditions

The planned research project is a monocentric, randomized controlled study conducted by the Heart and Brain Research Group—a cooperation project between the Department of Cardiac Surgery of the Kerckhoff Heart and Thoracic Center in Bad Nauheim and the Department of Neurology of the University Hospital Giessen. Our working group consists of members of the Departments of Neurology, Neuropsychology, Radiology, and Cardiac Surgery who are responsible for the study procedure, including preparing the protocol, monitoring the study, and writing the study reports. This study protocol follows the Standard Protocol Items: Recommendations for Interventional Trials (SPIRIT) guidelines (17). The data monitoring committee was not believed to be necessary, as no adverse effects of cognitive training are expected.

### Trial registration

The study is prospectively registered with ClinicalTrials.gov (Identifier: NCT04493996. First posted: July 31, 2020. First patient enrolled: August 14, 2020).

### Dissemination policy

Our aim is to make the study results available to the public, healthcare providers, and scientists by publication in the public press, at scientific congresses, and as original articles in peer-reviewed journals. The results will be reported regardless of the size and direction of the effect.

### Recruitment

A study coordinator will receive information from the Department of Cardiac Surgery of the Kerckhoff Heart and Thoracic Center in Bad Nauheim about planned elective cardiac surgeries. After screening inclusion and exclusion criteria, patients will be informed in detail about the purpose and procedure of the study project.

### Inclusion criteria

- Elective cardiac surgery (on pump)
  ° Coronary artery bypass surgery (CABG)
  ° Aortic valve replacement (AVR)
  ° Mitral valve replacement/reconstruction (MVR)
  ° Combination surgery (CABG+AVR, CABG+MVR, AVR+MVR, CABG+AVR+MVR)

- Age > 18 years
- Good knowledge of the German language (cognitive training and neuropsychological tests are language-dependent).

### Exclusion criteria

- Preexisting psychiatric disorders (depression and dementia) with acute clinical symptoms can impair psychological performance.
- Preexisting neurological disorders (stroke, Parkinson's disease, and multiple sclerosis) with acute clinical symptoms can impair psychological performance.

### Randomization

Randomization will be implemented using a computer-generated randomization list (www.randomization.com) with a 1:1 blocked allocation ratio and block sizes of 10. The randomization list will be generated, sequentially numbered, and concealed before the start of the study by the randomization list holder, who is located in the department of Neurology at the University Hospital Giessen and Marburg. After recruitment and the first preoperative neuropsychological examination, a study coordinator will allocate patients to the cognitive training or control group. A study coordinator will inform the psychological training supervisor about which patients should be trained.

### Blinding

Neuropsychologists and nursing staff who will be involved in the assessment of outcome variables will be blinded for randomization status. The training sessions will be conducted by a psychological training supervisor who is not involved in the evaluation of outcome parameters to ensure blinding. The holder of the randomization list is not involved in the supervision of the training or the evaluation of all examinations on neuropsychometrical functions and delirium. In addition, the randomization list and its holder are located in the department of Neurology at the University Hospital in Giessen where the psychological evaluations, the intervention, and the surgery do not take place. Furthermore, when we inform patients by mail about which randomization group they are assigned to, we instruct them for all subsequent assessments not to inform the investigators about their randomization status.

### Study process

After we obtain written consent from the patients, a detailed neuropsychometric assessment will be carried out 2–3 weeks preoperatively, and the patients will provide other information *via* questionnaires. These questionnaires, which must be completed by the patients themselves, refer to depression, anxiety, health-related quality of life, cognitive and social activities in everyday life, and questions on cognitive failures in everyday life. In addition, close relatives will also assess cognitive failures in the patients' everyday life in a separate questionnaire. Following neuropsychological assessment, randomization into a cognitive training group or control group will be performed. Patients in the training group will receive a package with the training material by mail. Those in the control group will receive a letter stating that they will not be involved in a training program. The control group will receive neither cognitive training nor placebo intervention. A second detailed neuropsychometric status assessment will be performed when patients are admitted to the acute clinic, which usually is scheduled 1 day before surgery. In addition, patients will complete questionnaires on depression, anxiety, and recent cognitive and social activities. At the time of discharge from the acute clinic, a short cognitive screening test (MOCA) will be carried out. Three months after surgery, all patients will be included in a final examination, which will take place at the Kerckhoff Clinic in Bad Nauheim, Germany, or at the patients' homes if required. This examination will include a further detailed neuropsychometric assessment, as well as questionnaires on depression, anxiety, health-related quality of life, questions on cognitive failures in everyday life, and recent cognitive and social everyday activities.

Baseline demographics and characteristics will include age, gender, years of educational and occupational background, body mass index, preexisting medical conditions (arterial hypertension, diabetes mellitus, dyslipidemia, and severity partition for left ventricular ejection fraction [defined by Lang et al. (18)]), heart failure, renal insufficiency (defined by a creatinine value above the in-house norms (men: >1.2 mg/dl, women: >0.9 mg/dl)), preoperative medication, type of surgery (CABG, AVR, MVR, and combination surgery), anesthesia and analgesics administered (type, amount, and duration), duration of the operation, duration of extracorporeal circulation, cross-clamp time, lowest body temperature, invasive ventilation time, peri- and postoperative complications (delirium, arrhythmia, atrial fibrillation, renal insufficiency, acute blood loss anemia, transient ischemic attack, stroke, dysarthria, aphasia, and death), days on the intensive care unit, and the total length of stay on the normal ward. We plan no systematic and standardized pre- or postoperative neuroradiological imaging.

**Figure 1:**
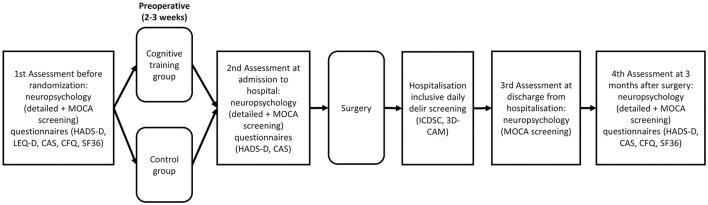
Timeline of the study-related events. The first examination includes neuropsychological tests and questionnaires before randomization and training. After the preoperative training period, neuropsychological tests and questionnaires will be administered a second time on admission to the acute clinic. After the surgery, daily delirium screenings will be performed and, on discharge from the acute clinic, neuropsychological screening will be carried out. To determine the long-term effects of cognitive training, neuropsychological tests and questionnaires will be carried out 3 months after surgery. MOCA, Montreal Cognitive Assessment; HADS, Hospital Depression and Anxiety Scale; LEQ-D, Lifetime of Experiences Questionnaire; CAS, Cognitive Activity Scale; CFQ, Cognitive Failure Questionnaire; SF-36, 36-Item Short Form Health Survey; ICDSC; Intensive Care Delirium Screening Checklist; CAM, Confusion Assessment Method.

The study process is shown in Figure 1. A detailed trial schedule according to the SPIRIT guidelines for study protocols is shown in Table 1 (17).

**Table 1:** Study plan for recruitment, interventions, and investigations.

**Time point**	***t_0_***	***t_1_***	***t_2_***	***t_3_***	***t_4_***	***t_5_***	***t_6_***	***t_7_***	***t_8_***
**Recruitment**
Inclusion criteria	X								
Informed consent	X								
Randomization		X							
**Interventions**
Surgery					X				
Cognitive training			X						
**Assessments**
MOCA	X			X				X	X
BVMT-R	X			X					X
VLMT	X			X					X
TMT	X			X					X
LNS	X			X					X
SKT-7	X			X					X
RWT	X			X					X
CFQ	X								X
LEQ-D	X								
CAS	X			X					X
HADS	X			X					X
SF36	X								X
ICDSC						X			
3-CAM							X		

MOCA, Montreal Cognitive Assessment; BVMT-R, Brief Visuospatial Memory Test-Revised; VLMT, Verbaler Lern- und Merkfähigkeitstest (Auditory Verbal Learning and Memory Test); TMT, Trail Making Test; LNS, Letter Number Span; SKT, Syndrom-Kurztest Test (Short Performance Test); RWT, Regensburger Wortflüssigkeits-Test (Regensburger Word Fluency Test); CFQ, Cognitive Failure Questionnaire; LEQ-D, Lifetime of Experiences Questionnaire; CAS, Cognitive Activity Scale; HADS, Hospital Depression and Anxiety Scale; SF-36, 36-Item Short Form Health Survey; ICDSC, Intensive Care Delirium Screening Checklist; CAM, Confusion Assessment Method.

### Primary outcomes

1. Number of participants with POCD at 3 months after surgery.
2. Number of participants with POCD at the time of discharge from the acute clinic.
3. Number of participants with POD during the stay in the intensive care unit.
4. Number of participants with POD during the stay in the normal ward.

### Secondary outcomes

1. Change from baseline cognitive failures in everyday life (CFQ) at 3 months after surgery.
2. Change from baseline health-related quality of life (SF-36) at 3 months after surgery.
3. Changes from baseline neuropsychological parameters at the end of cognitive training.
4. Number of participants with POCI at 3 months after surgery.
5. Number of participants with POCI at the time of discharge from the acute clinic.

### Planned statistical analyses

Postoperative cognitive decline is defined as a decline and POCI as an improvement from pre- to post-assessment of at least 1 SD in at least 20% of all neuropsychological subdomains (4). The difference of 1 SD between pre- and post-assessment will be measured using Z-scores, defined by the difference in individual raw values from the mean value of the total baseline data divided by the SD of the total baseline data. We will use the criteria of the *Diagnostic and Statistical Manual of Mental Disorders* (DSM-5) (19) to define neuropsychological subdomains, as shown in Table 2. As some neuropsychological parameters can be contextually grouped into cognitive subdomains (see Table 2), we will summarize them by the mean value. POCD/POCI at the time of discharge from the acute clinic is defined as a decrease or increase between pre-examination and post-examination of 1 SD within the total score of the MOCA screening test. POD is defined as the occurrence of at least one delirious episode during the stay in the intensive care unit or normal ward. POCD/POCI and POD will be compared with Pearson's chi-square test (or Fischer's exact test). The effect size will be reported by odds ratio (OR) with a 95% confidence interval (CI).

**Table 2:** Definition of neuropsychological parameters and cognitive subdomains.

**Cognitive domain (DSM-5)**	**Cognitive subdomains (DSM-5)**	**Neuropsychological parameter**	**Task (scale of measurement)**
Learning and memory	Visual immediate memory span	BVMT-R	Recalling objects immediately (number of correct items)
	Visual free recall	BVMT-R	Recalling objects after delay (number of correct items)
	Visual recognition memory	BVMT-R	Recognizing between learned and new objects (number of correct items)
	Verbal immediate memory span	VLMT	Recalling items of word list (number of correct items, first trial)
	Verbal free recall	VLMT	Recalling items of word list (summarized number of correct items, learning trials)
		VLMT	Recalling items of word list (number of correct items, short delay)
		VLMT	Recalling items of word list (number of correct items, long delay)
	Verbal recognition memory	VLMT	Recognizing between learned and new words (number of correct items)
Complex attention	Selective attention	TMT-A (speed)	Linking numbers in ascending order (seconds)
Executive functions	Verbal working memory	LNS	Mentally reorganization of letters and numbers (span)
	Cognitive flexibility	TMT-B (speed)	Linking numbers and letters in alternately order (seconds)
	Inhibition	SKT-7	Naming interfering letter, e.g., “S” instead of “T” (seconds)
Language	Word fluency	RWT (phonetic)	Naming words with specific initial letter (number of correct items)
		RWT (semantic)	Naming words from a specific category (number of correct items)
Perceptual motor	Visuo-construction	MOCA (3-D figure)	Drawing a 3-D figure (number of correct items)

BVMT-R, Brief Visuospatial Memory Test-Revised; VLMT, Verbaler Lern- und Merkfähigkeitstest (Auditory Verbal Learning and Memory Test); TMT, Trail Making Test; LNS, Letter Number Span; SKT, Syndrom-Kurztest Test (Short Performance Test); RWT, Regensburger Wortflüssigkeits-Test (Regensburger Word Fluency Test); MOCA, Montreal Cognitive Assessment.

Analyses of covariance (ANCOVAs) will be conducted to determine the effects of cognitive training on each neuropsychological parameter, CFQ, SF-36, and HADS-D. In the ANCOVA, the postoperative test value will be the dependent variable, groups will be the fixed factor, and the preoperative test value will be the covariate. Confounder variables that could affect the results will be implemented in addition to the preoperative cognitive values as further covariates to the ANCOVA. Confounder variables are defined as whether a correlation analysis among demographic variables, perioperative details, and neuropsychometrical changes between pre- and postoperative testing is significant.

Assumptions for ANCOVAs will be tested using a visual inspection of QQ and distribution plots of the dependent variable for normality, the Levene test for variance-homogeneity of the dependent variable, and a statistically significant correlation between the dependent variable and covariate (preoperative test value) calculated with Pearson's product–moment correlation. When assumptions for the ANCOVA are violated, difference values between pre- and post-tests will be calculated, followed by Mann–Whitney U-tests for between-subject effects. The effect sizes of continuous results will be given in η2.

If peri- and postoperative complications such as delirium, stroke, cardiovascular events, or other complications occur which could affect neuropsychological performance, and if these factors are unbalanced between the groups, we will calculate a subgroup analysis without those patients to reveal a more stringent outcome effect. In addition, we will perform subgroup analyses for each of the individual surgery cohorts, including AVR, MVR, CABG, and combination surgeries (AVR+MVR, CABG+AVR, CABG+MVR, and CABG+AVR+MVR) if the sample size of these subgroups allows for statistical analysis. Perioperative medical conditions or drug factors may have an impact on cognitive function. With the randomization principle, we expect these to be equally distributed between the groups. Otherwise, if the groups differ with respect to these factors, these can be accounted for in a subgroup analysis.

All analyses will be performed using the statistical software SPSS (version 22) and JASP (version 0.12.2).

Furthermore, interim analyses will be carried out during the investigation period to identify adverse events, overwhelming effects, or futility of the intervention arm. In this case, the study could be terminated before its planned completion. The decision will be made by the members of the study team.

### Power and sample size estimation

Because there are currently no effect sizes for extensive paper-and-pencil-based preoperative cognitive training in cardiac surgical patients using extracorporeal circulation, and because their influence on the incidence of POD and POCD is unclear, we have estimated sample size based on the effect sizes of general cognitive training in older healthy individuals, which ranges between d = 0.4 and d = 0.9 (20, 21). To not underestimate or overestimate the required sample size, we use an average value of d = 0.65. To find an effect size of d = 0.65 at a test power of 0.8 (alpha = 0.05) within an ANCOVA, a sample size of 77 subjects is required. With an estimated dropout rate of 23% in the preoperative test phase, the total sample size will be 100 subjects. The sample size per group is thus 50 patients. We used the analysis software G^*^Power-3 to calculate the sample sizes and the statistical power (22). We want to note that the power analysis refers to our secondary outcome “Changes from baseline neuropsychological parameters at the end of cognitive training.” Accordingly, the primary outcomes regarding the incidence of POD and POCD will be calculated exploratively.

### Metric assessments

Cognitive tests will be performed by a neuropsychologist 2–3 weeks before surgery, at admission to the acute clinic, during postoperative stays in intensive care and the normal ward, at discharge from the cardiac surgery clinic, and 3 months after surgery. Parallel test forms will be used in the follow-up examinations to account for individual learning effects. Parallel test forms will be counterbalanced for each test time point.

#### Objective cognition

The cognitive test battery measures verbal and visual memory with immediate-, free recall-, and recognition conditions, selective attention, verbal working memory, cognitive flexibility, inhibition, word fluency, and visuoconstruction.

With the German-validated version of the Montreal Cognitive Assessment (MOCA) (23), cognitive functions such as visuoconstructive ability, object naming, verbal memory, working memory, attention, phonematic word fluency, abstraction, cognitive flexibility, and orientation are assessed within the framework of a 10-min screening procedure (23).

Verbal memory will be assessed using the Verbaler Lern- und Merkfähigkeitstest (VLMT), a modified German version of the Rey Auditory Verbal Learning Test (24). This test can be used to evaluate immediate-, free recall-, and recognition conditions. Between the short-delayed and longer-delayed verbal episodic memory recall, non-verbal cognitive tests are performed to avoid possible effects of interfering words not included in the learned word list.

With the Letter–Number Test (LNS), the verbal working memory will be tested. The patient is supposed to rearrange a mixed sequence of letters and numbers through mental reorganization in such a way that first all numbers and then all letters are to be named in ascending order (25).

Visual memory will be examined with the Brief Visuospatial Memory Test-Revised (BVMT-R) (26). In the BVMT-R, the patient is shown six geometric figures for 10 s on a DIN A4 sheet of paper, which are to be drawn directly afterward. This procedure is repeated with the same figures in a total of three learning trials. The figures are to be freely replicated in a time-delayed episode with the following recognition help.

Selective attention will be examined using the Trail Making Test A (TMT-A), for which three validated parallel test versions are available (27). In the TMT-A, the patient has to connect numbers in ascending order on a test sheet as fast as possible.

Cognitive flexibility will be measured by the Trail Making Test B (TMT-B) (27) and cognitive inhibition by a subtest of the Syndrom-Kurz Test (SKT-7) (28). With the TMT-B, the patient's task is to connect numbers and letters alternately in ascending order. In the SKT-7, the patient has to rename a series of letters (e.g., “S” instead of “T,” and vice versa).

The semantic-categorical word fluency is tested with the Regensburger Wortflüssigkeits-Test (RWT) (29). In this test, the patient has to name in 1 min as many words as possible from a certain category.

#### Subjective cognition

Study patients will be asked to complete a validated German 25-item version of the Cognitive Failures Questionnaire for Self-Assessment (s-CFQ) (30). Because memory disorders play an important role in the everyday functions of patients, the s-CFQ was modified with four additional questions related to memory failures, taken from the validated German version of the Memory Complaint Questionnaire (31). Close relatives of the patients will also be asked to answer an eight-item cognitive questionnaire for external assessment (f-CFQ) with regard to the patients (32). All have to be answered on a 5-point scale from “never” to “very often.” The purpose of these questionnaires is to investigate the frequency of failure in daily living in terms of memory, attention, action, and perception in the past 3 months.

#### Cognitive reserve/cognitive everyday activities

The Lifetime of Experiences Questionnaire (LEQ-D), which has been translated and validated into German, is intended to determine the cognitive reserve in 34 items for three areas of life: education, occupation attainment, and lifestyle activities (33). These relate to early-, midlife-, and late-life reserve measures. Because the LEQ-D records the cognitive everyday activities unspecifically for the time immediately before surgery, the cognitive activity scale (CAS) according to Tow et al. (12) will also be used. This scale covers 12 cognitive activities, such as reading the newspaper, solving crosswords, writing, or group meetings, related to the previous week.

#### Delirium

Postoperative delirium will be examined during the stay in the intensive care unit with the German-validated Intensive Care Delirium Screening Checklist (ICDSC) (34). To be able to record the status of POD with high sensitivity and specificity, even during the stay in the normal ward, a newly developed version of the Confusion Assessment Method (3D-CAM) validated in German will also be used (35). Both tests record the clinical symptoms of consciousness, attention, orientation, hallucinations, psychomotor retardation or agitation, speech, and changing symptoms by observing behavior and asking concrete questions to the patient. The ICDSC also documents disturbances in the sleep–wake rhythm.

#### Depression and anxiety

Patients assessed their recent (prior week) depressive and anxiety symptoms using the validated German version of the Hospital Anxiety and Depression Scale (HADS-D) (36). Each scale contains seven questions to be answered by the patients.

#### Health-related quality of life

Health-related quality of life will be assessed with the 36-Item Short Form Health Survey (SF-36, Version 1.0) (37). The SF-36 includes 36 items covering eight health-related factors, including physical functioning (10 items), role limitations due to physical health (four items), role limitations due to emotional problems (three items), energy/fatigue (four items), emotional wellbeing (five items), social functioning (two items), pain (two items), and general health (five items). Furthermore, we will determine a total score across all eight factors, as well as a two-factor model, indicating the physical state of health (physical functioning, role limitations due to physical health, pain, and general health) and psychological state of health (role limitations due to emotional problems, energy/fatigue, emotional wellbeing, and social functioning). The answers provided by the patients within the factors refer to the last 4 weeks, except for the factor physical functions and the first question of the factor general health, which refer to the present state of health. Furthermore, it also contains a single item (item 2, health change), which indicates the extent to which the present health has changed in relation to the past year. The SF-36 will be scored using the RAND scoring method (38). Each item in the questionnaire is assigned a score from 0 to 100, with a higher score indicating a better health state.

For the CFQ, MCQ, and SF-36, missing data will be handled as follows. If the patients answered at least 50% of all items per factor and time point, the mean score of this factor will be calculated to determine the values of the factors. Items left blank (missing data) will not be considered. Therefore, the factor values will represent the average for all items of a factor that the respondent responded to.

### Data management

All personal data about recruited patients will be subject to medical confidentiality. Paper-based assessment forms will be used to record all variables. The data will be entered into an electronic database, which will be password-protected and double-checked for accuracy. All signed informed consent forms, assessment forms, and the randomization list will be stored in locked cabinets. Each patient will receive a sequential number recorded on the paper-based assessment forms and in the electronic database. Personal data such as first name, last name, and address will not be included in the paper-based assessment forms or the electronic database.

### Cognitive training

We have validated a postoperative paper-and-pencil-based cognitive training program for older cardiac-surgery patients with the effect of reduced POCD at discharge from rehabilitation (after 3 weeks of training) and at a 3-month follow-up (39). In the INCORE study, we will use this training program for the preoperative setting. Here, we will provide information about the concept of our training.

We derived the design of our cognitive training program using the German language-validated, paper-and-pencil-based intervention by Müller et al. (40). This program is intended to train working memory, cognitive flexibility, word fluency, and planning skills. To achieve better patient acceptance, we only adopted exercises that we found most useful and combined them with new tasks developed by our group. Furthermore, we initially constructed our training to address several cognitive functions that are particularly important in everyday life to maintain social functions and earning capacity. These include word fluency, working memory, attention, and the ability to plan. One training unit will last approximately 40 min and will be conducted for 2–3 weeks, about 6 days a week. The daily training program consists of eight different types of standardized tasks related to the processing of words, categories, images, mental arithmetic, and planning. On each training day, new words, categories, images, head calculations, and planning tasks will be presented. Each task takes between 2 and 10 min to complete. To manage their working time, the patients must use a digital clock. If the patient has any questions about the tasks, they can contact the training supervisor by telephone. In addition, the training supervisor will call the patient once a week to monitor cooperation. Each task contains precise written instructions that may be helpful in the execution. Patients will also be told that their exercise solutions will not be corrected. Therefore, it does not matter whether the solutions are right or wrong. In this way, we can avoid any possible pressure to perform and patients looking for the right solutions at home. The different types of tasks are presented in the following standardized order.

#### Phonetic word fluency

The patient receives three letters on a sheet of paper. The task is to note as many words as possible that begin with these letters within 2 min. This task is mainly used to train word fluency and was adapted from the work of Müller et al. (40).

#### Categorical word fluency

In this task, the patient receives three different categorical terms on a sheet of paper. The task is to find and note as many words as possible within 2 min that can be assigned to these categories. This task serves mainly to train word fluency and was adapted from the work of Müller et al. (40).

#### Picture stories

The patients receive four to five popular German picture stories by German illustrators such as Wilhelm Busch, Erich Ochser, or Hans Juergen Press, with 3–16 pictures of a story in mixed order. Within 5 min, the pictures have to be arranged mentally in a meaningful order. The newly invented order is to be documented by numbering the pictures with a pen. This task is mainly intended to train working memory and was created by our group.

#### Mental arithmetic

The patient is asked to perform several calculation tasks on one sheet of paper. The result of a first arithmetic problem involving the addition, subtraction, and multiplication of numbers must be memorized. The second step is to solve another arithmetic problem and memorize the result. In the last step, the last result should be subtracted from the first result, and the final result should be written down. The time limit for this exercise is 5 min. This task is mainly intended to train working memory and was adapted from the work of Mueller et al. (40).

#### Synonymic fluency

The next worksheet contains three different terms. For each term, patients must find words with similar meanings (synonyms). For example, if the term is wallet, then other words with similar meanings would be portemonnaie or money purse. The time limit is 2 min. This task is mainly intended to train word fluency and was created by our group.

#### Gap text

In the next training task, short stories will be presented. These are generally known stories by Wilhelm Busch, the Brothers Grimm, or Hans Christian Andersen or ancient German, Buddhist, and Japanese fables. The stories have gaps that are to be filled in with a self-chosen, meaningful word. The time limit for this exercise is 5 min. This task is mainly intended to train word fluency and working memory and was developed by our group.

#### Where is Waldo

An illustration of Martin Handford's “Where is Waldo?” is shown on a DIN A3 sheet. The picture contains dozens or more people doing a variety of things in a particular place. The task is to find specific people or objects listed on a sheet of paper by marking them with a pen on the illustration within 5 min. This task is mainly intended to train selective attention and working memory and was created by our group.

#### Organizing and planning

In the last task, the patient must read a text in which an imaginary person must perform certain actions or organize appointments. The patient's task is to solve the problems and write down the solutions on a sheet of paper. The time limit for this task is 10 min. The task primarily serves to train planning ability and working memory and was adapted from the work of Müller et al. (41).

To control the quality of the cognitive training, we will assess the number of tasks completed by the patients and report it as a percentage.

## Discussion

This research project offers our patients preoperative cognitive training based on paper-and-pencil aiming to prevent POD and POCD.

Investigations have already been conducted to treat the preoperative physical condition of cardiosurgical patients to improve postoperative outcomes, which is defined by the term prehabilitation (42). Initial efforts have also been made with regard to preoperative cognitive training. Saleh et al. showed a lower incidence of POCD 1 week after gastrointestinal tumor resection with a controlled 3 x 1-h preoperative memory strategy training (loci method) (43). Several studies have investigated the effect of perioperative computer-based cognitive training on postoperative cognition after cardiac surgery. In the study conducted by O'Gara et al. (44), the training was performed at least 10 days before surgery up to 4 weeks postoperatively, which had no significant effect on the frequency of POD or POCD. Humeidan et al. found a reduced incidence of POD with preoperative computer-based cognitive training (median 4.6 h) (45). Vlisides et al. showed that participation in preoperative computer-based cognitive training was difficult for older adults who underwent surgery (46). Mainly because of the feeling of overwhelming demands and technical problems with the 7-day computer exercises, 48% of the patients discontinued the training.

The difference in our study concept compared with the above-mentioned studies is that we use paper-and-pencil-based instead of computer-based cognitive training, which is probably more feasible and effective for older patients. Furthermore, our program will take place in the preoperative phase, which will help to address differences between preoperative and [as we have conducted in the past; (39)] postoperative training. As we saw in the postoperative setting, this cognitive training appears to be adequately feasible regardless of educational status. Therefore, we did not set a limit for a required educational status in the inclusion criteria.

Cognitive training allows patients to be independent, responsible, and active. With the knowledge that cognitive deficits can be actively prevented, anxiety regarding a severe operation can be reduced. This has particular clinical relevance, as preoperative anxiety is considered a risk factor for increased morbidity and mortality in cardiac surgery patients (47).

Morphological alterations have also been found in response to cognitive training in healthy adults with reproducible increased patterns in the structure of gray and white matter (48) and patients with memory impairments, with increased volume of gray matter in certain brain regions (49). These effects are mainly attributed to cognitive and neuronal plasticity.

The limitations of this study are as follows. The study only includes patients undergoing cardiac surgery with ECC. A comparison with patients undergoing off-pump cardiac surgery, non-cardiac surgery, or non-surgery (healthy controls) is, therefore, not intended. A placebo intervention for the control group is intentionally avoided because the cognitive effects of placebo interventions on cognitive performance are hardly controllable. To convincingly communicate to patients that the placebo intervention could have an influence on their memory and thus also achieve a willingness to participate, the structure of the placebo intervention would have to be closely related to cognitive training (e.g., crossword puzzles, conversation therapy, computer games, cognitive information, etc.) and would thus also achieve cognitive training effects. This could formally affect the quality of the study and the transferability of the results. As we do not know whether paper-and-pencil-based preoperative cognitive training can reduce the incidence of POD and POCD, we do not see any ethical problems if the control group does not receive cognitive training. Finally, in principle, it may be that cognitive intervention proves useless and that patients' preoperative time would be better spent on other validated and clinically relevant interventions. Furthermore, according to our study design, it is not possible to put the patients of the control group on a waiting list, as they would have already been operated on. If a subgroup analysis for each cardiac surgery method (CABG, AVR, MVR, and combination surgeries such as AVR + MVR, CABG + AVR, CABG + MVR, and CABG + AVR + MVR) is statistically inadequate due to the small sample size, we cannot adequately perform generalization of the training effect on a single surgery cohort.

In addition to altering preoperative cognitive performance to potentially counteract POD and POCD, other risk factors regarding POD such as poor sleep burden (50) and heart rate response/recovery to exercise (51) can also be modified in the preoperative setting and may be linked to preoperative risk factors such as depression and reduced cognitive functions.

Should it become evident that the use of our cognitive training can both reduce the incidence of POCD and POD and improve health-related quality of life, one possibility could be to integrate this intervention into a standardized prehabilitation program. It can also be evaluated in other patient populations affected by postoperative neurocognitive dysfunctions.

## Trial status

The study is currently enrolling patients. This study is registered with ClinicalTrials.gov (Identifier: NCT04493996. First posted: July 31, 2020. The first patient was enrolled on August 14, 2020). Recruitment is expected to be completed in November 2023. Protocol version: 1.0 (17-01-2022).

## Ethics statement

The studies involving human participants were reviewed and approved by by the Ethics Committee of the Justus Liebig University Giessen (Ref.: 48/20). Written informed consent will be obtained from the patients to participate in this study. All changes to the study protocol will be submitted to the Ethics Committee.

## Author contributions

MB: conceptualization, data curation, formal analysis, funding acquisition, investigation, methodology, project administration, supervision, and writing the original draft. RM: data curation, investigation, project administration, and review and editing. TG: methodology, funding acquisition, project administration, and review and editing. GS: methodology, supervision, and review and editing. JD: data curation, project administration, and review and editing. JE-S: methodology, reviewing, and editing. TD: reviewing and editing. Y-HC: project administration, resources, and reviewing and editing. MS and MJ: methodology, funding acquisition, project administration, resources, and reviewing and editing. All authors contributed to the article and approved the submitted version.
